# Design and structural characterization of autoinhibition-compromised full-length Ran

**DOI:** 10.1038/s41392-020-00398-y

**Published:** 2021-02-03

**Authors:** Yuping Tan, Yuqing Zhang, Qiao Zhou, Da Jia, Qingxiang Sun

**Affiliations:** 1 Department of Pathology, State Key Laboratory of Biotherapy and Cancer Centre, West China Hospital, Sichuan University and Collaborative Innovation Centre of Biotherapy, Chengdu, 610041 China; 2 Key Laboratory of Birth Defects and Related Diseases of Women and Children, Department of Paediatrics, Division of Neurology, West China Second University Hospital, Sichuan University, Chengdu, 610041 China

**Keywords:** Structural biology, Isolation, separation and purification

**Dear Editor**,

The Ras-related nuclear protein Ran is a small GTPase that functions in nuclear transport, mitotic spindle formation, nuclear-envelope/nuclear-pore complex assembly, and other diverse cytoplasmic activities.^1,2^ Unlike other Ras superfamily proteins, Ran contains a unique autoinhibitory C-terminal tail (C-tail) that accounts for an estimated tenfold lower affinity for GTP as compared with GDP. Multiple missense cancer mutations at the C-tail have been observed, but the biological significance is unknown. The study of recombinant Ran proteins often helps provide insights into cellular and disease mechanisms. To purify active Ran (GTP or GTP analogue-bound), one could either mutate the catalytic residue Q69 to slow down the intrinsic GTP hydrolysis or charge the protein with an excess of GTP analogue. These methods are complicated, inefficient, and prone to protein denaturation. Another strategy is to delete the C-terminal 37 residues or only the DEDDDL terminus. However, these mutants are of limited usage due to their inability to bind to key regulators, such as RanBP1 (Ran-binding protein 1).

To generate more broadly applicable GTP-bound proteins, and to set a foundation for investigating cancer mutations, four mutations were designed to disrupt the interaction between the C-tail and the G-domain (Fig. 1a). In the RanGDP crystal structure, C-terminal residues L182, M189, and Y197 are each inserted into a surface cavity on the G-domain, and the C-terminal helix is intimately packed against G-domain residue A133. A133D, L182A, M189D, and Y197A were designed to disrupt these interactions. Among these residues, A133 and L182 are strictly conserved from fungi to humans (Fig. 1b). Together with Ran^WT^, Ran^Q69L^ (unable to hydrolyse GTP), Ran^1–179^ (without the C-terminal 37 residues), and Ran^1–210^ (without the DEDDDL C-terminus), these proteins were purified from *Escherichia coli*. The designed mutants were similar to Ran^WT^ in size exclusion chromatography (single peak) and purification yields, and more stable than Ran^1–179^ (Fig. 1c). The C-tail shields a large hydrophobic area in RanGDP. Unlike 1-179, the dislodged and flexible C-tail of GTP-bound WT or mutant Ran remains able to inhibit hydrophobic contacts and prevent protein aggregation.

**Fig. 1:**
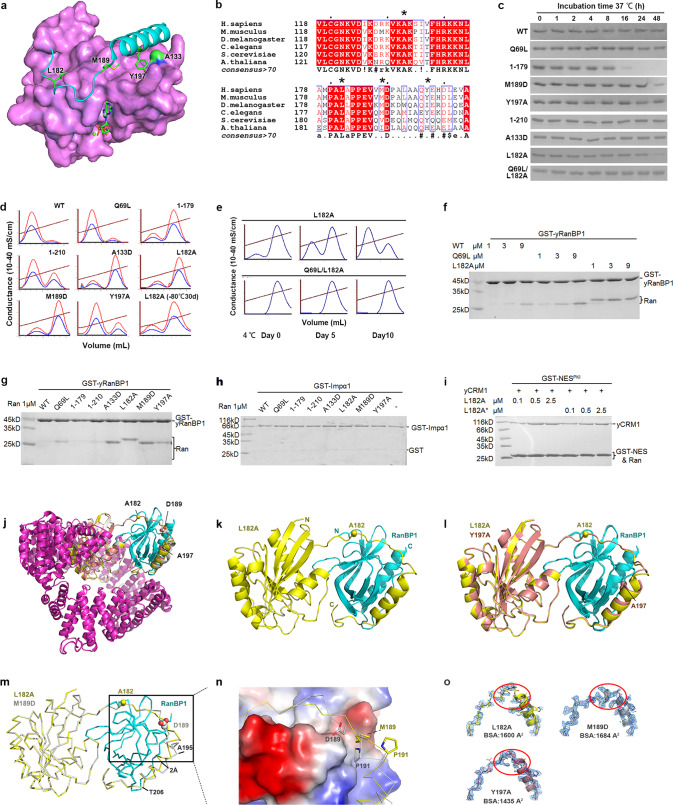
Design and characterization of GTP-bound full-length Ran via the disruption of the autoinhibitory C-terminal tail. **a** An illustration of the mutation sites. The Ran (3GJ0) tail is displayed as a cyan cartoon. The G-domain is covered in a partially transparent magenta surface. Four loci of mutagenesis (L182, M189, Y197, and A133) are shown as green sticks or surface. None of these residues is in direct contact with GTP (green sticks) or Mg^2+^ (green sphere). **b** Multiple sequence alignment of Ran from distant species, with the consensus displayed at the bottom. Mutated residues (A133, L182, M189, Y197) are indicated with an * above the alignment. **c** Stability of different Ran proteins. Ran proteins (1 µM) were incubated at 37 °C for different lengths of time in a buffer containing 20 mM Tris pH 7.5, 300 mM NaCl, 10% glycerol, 5 mM MgCl_2_, 2 mM DTT, 0.001% Triton-X 100, and 50 µM GTP. After thorough spin, the supernatants were analysed by SDS-PAGE. These mutations are not cancer-derived. **d** Q anion-exchange analysis of bound nucleotide in the purified Ran proteins. GDP and GTP were eluted as the first and second peaks, respectively. A260, A280, and conductivity are shown as red, blue, and brown lines, respectively. Y197A is less GTP-bound as compared with the other three designed mutants. This is likely due to its higher flexibility (B-factors) and weak G-domain binding energy contribution. **e** Bound nucleotide status of L182A and Q69L/L182A at 4 °C in a buffer containing 20 mM Tris pH 8.0, 100 mM NaCl, 5 mM MgCl_2_, and 5 mM β-mercaptoethanol. **f** GST-tagged yeast (*Saccharomyces cerevisiae*) RanBP1 (GST-yRanBP1) pull-down of different concentrations of Ran^WT^, Ran^Q69L^, and Ran^L182A^. Ran^L182A^ consistently appears larger than the rest of Ran mutants in SDS-PAGE, although the sequence has been verified by DNA sequencing. **g** GST-yRanBP1 pull-down of Ran and different mutants at 1 µM concentration. Ran^1-179^ is not supposed to bind RanBP1 even if it is GTP-bound. The same batch of freshly purified Ran proteins was used here. **h** GST-Impα1 (71-529) pull-down to show that the binding to RanBP1 is specific. Since Ran and GST are similar in size on SDS-PAGE, GST-Impα1 rather than GST was used in this pull-down. **i** GST-NES pull-down of yCRM1. L182A purified in the presence of 1 mM GTP is denoted as L182A*. There is no significant CRM1 binding difference for RanL182A purified in the presence or absence of GTP. **j** Superimposition of three Ran-RanBP1-CRM1 crystal structures obtained in this study. CRM1 is shown in magenta, RanBP1 is shown in cyan, and Ran is shown in yellow (L182A), grey (M189D) and salmon (Y197A). Mutated residues are shown as spheres. CRM1 was used to help the crystallization process, but was omitted for clarity in the subsequent panels since it is distant from the mutation sites. **k** Crystal structure of Ran^L812A^ (yellow) in complex with yRanBP1 (cyan). The mutated residue L182 (sphere) does not interact with RanBP1. **l** Superimposition of Ran^L182A^-RanBP1 with Ran^Y197A^-RanBP1. **m** Superimposition of Ran^L182A^-RanBP1 with Ran^M189D^-RanBP1. To improve clarity, this panel is shown in ribbon representation. **n** Zoom-in view of the boxed region in ‘panel-**m**’, with RanBP1 displayed in the electrostatic surface potential map. Residues 189 and 191 are shown in stick representation. **o** 2F_o_-F_c_ omit maps (blue mesh) of Ran C-tail contoured at 1*σ* level. Ran is shown as cartoon and stick representation. The electron density of Ran^M189D^ is improved in the circled region. The buried surface area (BSA) between Ran C-terminal residues (177-216) and RanBP1 was calculated by PISA in CCP4 and is displayed in the figure. The buried surface area for Ran^WT^ is 1591Å^2^

The purified proteins were over 90% nucleotide-bound, though no nucleotides were added in the purification stages. To determine the status of Ran-bound nucleotides, the proteins were denatured using 100 mM NaOH, and the samples were analysed using a Q-sepharose anion exchange column (Fig. 1d). As expected, Ran^WT^, Ran^1–179^, and Ran^1–210^ were 6, 87, and 35% GTP-bound, respectively. Though Ran^Q69L^ does not hydrolyse GTP, only 12% was GTP-bound. Notably, the C-dis (C-tail disrupting) mutants Ran^A133D^, Ran^L182A^, and Ran^M189D^ were substantially GTP-bound (79-85% GTP). Only 23% of Ran^Y197A^ was loaded with GTP, suggesting that Y197 is less important for autoinhibition.

Although the intrinsic GTP hydrolysis of Ran is slow, a reduced level of bound GTP was observed for Ran^L182A^ after ten days of storage at 4 °C (Fig. 1e). To prevent intrinsic GTP hydrolysis, a Q69L/L182A double mutant was generated, which exhibited 100% GTP binding after purification. In the absence of free nucleotides at 4 °C, this double-mutant remained 100% GTP-bound for at least ten days without obvious precipitation. After one month of storage at −80 °C, Ran^L182A^ was similarly GTP-bound as freshly purified Ran^L182A^ (Fig. 1d).

RanBP1, which relieves the blockage of RanGTP hydrolysis by karyopherins, displays a high affinity for RanGTP but not RanGDP. RanBP1 pull-down was used to check the binding activity of freshly purified mutants. Unlike Ran^WT^ and Ran^Q69L^, which exhibited dose-dependent binding, Ran^L182A^ bound to RanBP1 at all concentrations (Fig. 1f). When all Ran mutants were tested at a low concentration, the C-dis mutants were more bound to RanBP1 than the rest of the proteins (Fig. 1g), in agreement with the greater fraction of bound GTP. The binding to RanBP1 by different mutants was specific (Fig. 1h).

RanGTP forms a nuclear export complex with CRM1 (Exportin-1) and NES cargoes (nuclear export signal-containing proteins) in the nucleus. In pull-downs with CRM1, L182A dose-dependently enhanced CRM1 binding to GST-tagged NES (Fig. 1i). Further, no CRM1-binding difference was detected for parallelly purified Ran^L182A^ in the presence or absence of free GTP, suggesting that supplementing GTP in the purification buffers may not be necessary.

Because the C-tail of Ran is involved in RanBP1 binding, three C-terminal mutants (Ran^L182A^, Ran^M189D^, and Ran^Y197A^) were crystallized in complex with RanBP1 and CRM1 to examine whether these mutations alter RanBP1 binding (Fig. 1j, Supplementary Table 1). Since the L182 side chain does not directly contact RanBP1 (Fig. 1k), the differences between the L182A and WT structures (4HAT)^3^ are likely caused by differences in the NES groove, but not the mutation. One evidence is that the C-tail of Ran^Y197A^ is also similar to that of Ran^L182A^ (Fig. 1l). However, the C-tail of Ran^M189D^ exhibits substantial local changes (Fig. 1m). In the Ran^L182A^ structure, M189 is loosely packed on the edge of a hydrophobic pocket in RanBP1 (Fig. 1n). In Ran^M189D^, D189 (being more hydrophilic) is flipped out towards the solvent, and a previously solvent-exposed proline (P191) is inserted into that hydrophobic pocket. The movement of P191 drags towards the pocket a one-turn helix (A192–A195), which was originally part of a longer helix (A192–T206). The adjacent end of the now-much-shorter helix is shifted about 2 Å from its original position (Fig. 1m). It should be noted that this change is not due to crystal packing. Both the C-tail electron density and the RanBP1 contact area for Ran^M189D^ are increased as compared to the other mutants (Fig. 1o), suggesting possibly tighter RanBP1 binding. The observed binding changes reflect higher protein dynamics at the binding interface, as seen in other small GTPase interactions.^5^

The designed C-dis mutants simultaneously possess two advantages: (1) C-dis mutations enable the effortless and cost-effective purification of highly GTP-bound Ran. Via simple mutation to disrupt the autoinhibitory C-tail, RanGTP could be easily purified without adding any GTP or GTP analogues, nor performing the previously necessary GTP-charging steps. (2) The C-dis mutants are more stable than 1–179, and are able to bind to RanBP1 and RanBP2. The double-mutant Ran^Q69L/L182A^ was suitable for conducting isothermal titration calorimetry experiments, allowing for the determination of the exact RanGTP concentration. Using Ran^Q69L/L182A^ in an experiment could prevent the problem of RanGDP contamination.

In summary, we successfully designed and characterized four Ran mutants with greater levels of bound GTP by disrupting the C-tail. The designed mutants should be useful for a broad range of future experiments. Our unpublished data indicate that this type of mutation exists in cancers and may be pathogenic. Though the C-tail is unique to Ran, other small GTPases might also harbour mutations (besides the known Ras mutations, such as G12D) that perturb their GTP/GDP ratio. In line with this speculation, reports have shown that the C-terminal hypervariable region of K-Ras4B stabilizes its GDP binding, and cancer mutations could weaken this interaction.^4^ Mutations perturbing the GTP/GDP ratio may influence the activity of the GTPase, the transduction of signals, and eventually the fates of cells.^5^

## Supplementary information

Supplementary Materials

## Data Availability

Structure factors and atomic coordinates were deposited into the Protein Data Bank with the accession codes 5YRO, 5YTB, and 5YST.
