# Supplementary material for: Design and structural characterization of autoinhibition-compromised full-length Ran
Source: Signal Transduct Target Ther. 2021 Feb 3;6:44. doi: 10.1038/s41392-020-00398-y (PMC7854590; doi:10.1038/s41392-020-00398-y)
Supplement: Supplementary file 1 — Supplementary Materials [file 41392_2020_398_MOESM1_ESM.pdf]

Supplementary Materials for

Design and structural characterization of autoinhibition-compromised full-length Ran

Yuping Tan[1#], Yuqing Zhang[1#], Qiao Zhou[1], Da Jia[2], Qingxiang Sun[1*]

Correspondence to: qingxiang.sun@scu.edu.cn

This PDF file includes:

Materials and Methods
Table S1

Materials and Methods

Cloning, protein purification, structure determination

The human Ran mutants were cloned separately into a pET-15b expression vector incorporating an N-terminal 6x his-tag fusion. NES^{PKI}, IBB (1-70 amino acids of Importin α1) and the full length of yRanBP1, yCRM1, Imp β1, and Imp α1 were cloned into pGEX-4T-1 with an N-terminal GST tag and a tobacco etch virus (TEV) protease recognition linker. Expression of 6x his-Ran was induced by the addition of 0.5 mM isopropyl β-D-1-thiogalactopyranoside (IPTG) in *E. coli* BL21 (DE3) cells for four hours at 37˚C in LB Broth (Miller). Cells were harvested and sonicated in a buffer containing 20 mM Imidazole pH 8.0, 400 mM NaCl, 5 mM $MgCl_2$ and 1 mM PMSF. Proteins were purified on a Ni-NTA column and eluted in a buffer containing 300 mM Imidazole pH 8.0, 300 mM NaCl, 5 mM $MgCl_2$ and 1 mM beta-mercaptoethanol (BME). The proteins were purified on a Superdex 200 increase gel filtration column on Äkta Pure (GE Healthcare) using a buffer containing 20 mM Tris pH 8.0, 100 mM NaCl, 5 mM $MgCl_2$, 5 mM BME. Purified proteins were concentrated to 5-10 mg/mL using a Vivaspin turbo 15 (Sartorius) concentrator, and store at -80 ˚C. The GST-tagged proteins were purified as previously reported. Protein crystallization and structure determination is the same as reported earlier (reference 3). The data collection and refinement statistics were provided in Table S1.

GTP/GDP quantification

Proteins (500 µg, in less than 1 mL volume) were denatured by adding 100 mM NaOH at room temperature for 5 minutes. The denatured samples were added with 10 ml of buffer A (10 mM Tris pH 8.0) to reduce the ionic strength. The samples were loaded onto a Hitrap Q anion exchange column (GE Healthcare) and eluted with an increasing gradient of buffer B (1 M NaCl) on Äkta Pure (GE Healthcare). The peak areas were integrated using the Unicorn software.

Pull down assay

To assess different interactions, GST-tagged proteins were immobilized on GSH beads, and an immediate wash step was performed to remove unbound GST tagged proteins. Soluble proteins at indicated concentrations were incubated with the immobilized proteins in a total volume of 1 mL for one hour at 4 ℃ with gentle rotation. After three wash steps, bound proteins were separated by SDS PAGE and visualized by Coomassie Blue staining. The pull down buffer contained 20 mM Tris pH 7.5, 200 mM NaCl, 10% glycerol, 2 mM $MgCl_2$, 0.001% Triton-X100, and 2 mM DTT.

Table S1. Crystal data collection and refinement statistics.

	yCRM1:hRan^{L182A}:yRanBP1	*yCRM1:hRan^{M189D}:yRanBP1*	*yCRM1:hRan^{Y197A}:yRanBP1*
Cell axial lengths (Å)	*a*=*b*=105.26, *c*=307.42<br>α=β=γ=90	*a*=*b*=104.88, *c*=307.82<br>α=β=γ=90	*a*=*b*=105.29, *c*=305.73<br>α=β=γ=90
Spacegroup	$P4_32_12$	$P4_32_12$	$P4_32_12$
**Data collection**			
Resolution range (Å)	50.00-2.40 (2.44-2.40)	50.00-2.04 (2.08-2.04)	50.00-2.30 (2.34-2.30)
Number of observed reflections	583240 (28773)	1425362 (70962)	1021220 (46628)
Number of unique reflections	65435 (3233)	110107 (5417)	77397 (3822)
Completeness (%)	94.8 (95.4)	100.0 (100.0)	100.0 (100.0)
Redundancy	9.0 (8.9)	12.9 (13.1)	13.2 (12.2)
R_{pim}	0.070 (0.855)	0.028 (0.314)	0.058 (0.766)
Highest shell CC1/2	0.410	0.303	0.841
Mean I/I_{sigma}	8.4 (0.69)	13.4 (0.78)	27.4 (2.4)
Solvent content (%)	52.3	52.4	52.1
**Refinement**			
Resolution range (Å)	40.5-2.40 (2.43-2.40)	49.70-2.04 (2.06-2.04)	40.3-2.30 (2.33-2.30)
Number of working reflections	61682 (2430)	104507 (3305)	72671 (2591)
Number of test reflections	3319 (145)	5422 (168)	3853 (130)
R_{work}^{a}	0.207 (0.341)	0.196 (0.338)	0.191 (0.274)
R_{free}^{b}	0.245 (0.385)	0.224 (0.354)	0.228 (0.325)
R.m.s. deviation bond lengths (Å)	0.011	0.004	0.010
R.m.s. deviation bond angles (°)	1.164	0.732	1.013
Average B-factors (Å2) (# of atoms)	68.9 (11205)	50.3 (11609)	61.6 (11174)
**Ramachandran plot**			
Most favoured regions (%)	93.8	93.9	93.7
Allowed regions (%)	5.9	5.8	5.9
General allowed regions (%)	0.2	0.1	0.2
Disallowed regions (%)	0.1	0.2	0.2

$R_{work}^{b} = \Sigma|Fo - Fc|/|Fo|$, where Fc and Fo are the calculated and observed structure factor amplitudes, respectively

R_{free}^{c} calculated as for R_{work} but for 5.0% of the total reflections chosen at random and omitted from refinement for all data sets
